# Efficacy of Ethanol Extract of *Fructus lycii* and Its Constituents Lutein/Zeaxanthin in Protecting Retinal Pigment Epithelium Cells against Oxidative Stress: *In Vivo* and *In Vitro* Models of Age-Related Macular Degeneration

**DOI:** 10.1155/2013/862806

**Published:** 2013-09-12

**Authors:** Xinrong Xu, Li Hang, Binglin Huang, Yuanhua Wei, Shizhong Zheng, Wei Li

**Affiliations:** ^1^Department of Ophthalmology, Affiliated Hospital of Nanjing University of Traditional Chinese Medicine, 155 Hanzhong Road, Nanjing 210029, Jiangsu, China; ^2^Department of Ophthalmology and Otorhinolaryngology, The First Clinical Medical College, Nanjing University of Traditional Chinese Medicine, Nanjing 210046, China; ^3^Department of Ophthalmology, Affiliated Hospital of Jiangxi University of Traditional Chinese Medicine, Nanchang 330006, China; ^4^Department of Clinical Laboratory, Jiangsu Province Hospital of Traditional Chinese Medicine, Nanjing 210029, China; ^5^Collage of Pharmacy, Nanjing University of Traditional Chinese Medicine, Nanjing 210029, China

## Abstract

Age-related macular degeneration (AMD) is a major cause of blindness worldwide. Oxidative stress plays a large role in the pathogenesis of AMD. The present study was to evaluate the effects of *Fructus lycii* ethanol extract on AMD in mice and to investigate whether combination of lutein and zeaxanthin, two carotenoid pigments in *Fructus lycii*, could protect human retinal pigment epithelial ARPE-19 cells treated with hydrogen peroxide (H_2_O_2_) *in vitro*. We found that severe sediment beneath retinal pigment epithelium and thickened Bruch membrane occurred in AMD mice. However, *Fructus lycii* ethanol extract improved the histopathologic changes and decreased the thickness of Bruch membrane. Furthermore, the gene and protein expression of cathepsin B and cystatin C was upregulated in AMD mice but was eliminated by *Fructus lycii* ethanol extract. Investigations * in vitro* showed that ARPE-19 cell proliferation was suppressed by H_2_O_2_. However, lutein/zeaxanthin not only stimulated cell proliferation but also abrogated the enhanced expression of MMP-2 and TIMP-1 in H_2_O_2_-treated ARPE-19 cells. These data collectively suggested that *Fructus lycii* ethanol extract and its active components lutein/zeaxanthin had protective effects on AMD *in vivo *and * in vitro*, providing novel insights into the beneficial role of *Fructus lycii* for AMD therapy.

## 1. Introduction

Age-related macular degeneration (AMD) represents a progressive chronic disease of the central retina and is a leading cause of vision loss worldwide. The cause of AMD is complex, and many risk factors have been implicated including age, genetics, diet, and other environmental risk factors. Most of the visual loss occurs in the late stages of the disease due to one of two processes: neovascular AMD (wet AMD) and atrophic AMD (dry AMD) [1]. The recent few decades have witnessed advances in the treatment of wet AMD. Antiangiogenic agents targeting choroidal neovascularization such as pegaptanib, bevacizumab, and ranibizumab have shown a therapeutic promise for wet AMD [2–4]. Unfortunately, there is currently no proven treatment for dry AMD in the clinical context. 

Increasing understanding of the pathogenesis of AMD reveals that cathepsin B and cystatin C have important functions in the catabolism of outer membranous disc of visual cells. Cathepsin B is a thiol-dependent lysosomal proteinase that can degrade collagens, connective tissue proteins, and certain native enzymes [5]. Visual cells also secrete cystatin C, resulting in protection of the surface proteins from degradation. More recently, epidemiological evidence shows that cystatin C is associated with increased risk of developing exudative AMD [6]. Furthermore, matrix metalloproteinases (MMPs) and tissue inhibitor of metalloproteinases (TIMPs) produced by retinal pigment epithelium (RPE) cells are critically involved in maintaining the homeostasis of matrix components in the eye tissues [7]. Currently, attention has been increasingly addressed to these molecular events previously not considered in the context of RPE-driven mechanisms of AMD pathogenesis. 

The eye is an exceptional organ because of its continuous exposure to environmental chemicals, radiation, and atmospheric oxygen. There is a general consensus that cumulative oxidative damage is responsible for aging and may, therefore, play an important role in the pathogenesis of AMD [8]. Oxidative stress may cause injury to RPE, Bruch membrane, and choroid, which are layers in the eye involved in the pathophysiology of AMD. Antioxidant strategy has been proposed and tested in the treatment of dry AMD [9]. Recent studies show that hydroquinone, a major prooxidant in cigarette smoke and atmospheric pollutants, induces actin reorganization and bleb formation involved in sub-RPE deposits formation relevant to the pathogenesis of AMD [10]. There is evidence from observational studies suggesting that people who eat a diet rich in antioxidant vitamins (carotenoids, vitamins C and E) may be less likely to develop AMD [11]. Current pharmacological studies demonstrate that *Fructus lycii*, a well-known tonic medicine and a long-term healthy food without side effects in Asia, has predominant antioxidative and antiaging effects and can improve immunity [12]. In addition, the *β*-carotenes contained in this herb are important visual pigments and potent antioxidants. Of note, lutein and zeaxanthin, two common carotenoids in *Fructus lycii*, are constituents of the yellow macular pigment in the human retina [13]. These two compounds were also reported to exert neuroprotective effects by the mechanism of antioxidative stress in vision-threatening diseases, such as innate retinal inflammation, diabetic retinopathy, and light-induced retinal degeneration [14, 15]. However, the biological functions of these macular pigments are not fully understood. The current study was to investigate the *in vivo* effects of *Fructus lycii *ethanol extract on AMD induced by high-fat diet and hydroquinone intake in mice and to evaluate the* in vitro* effects of lutein combined with zeaxanthin on hydrogen peroxide- (H_2_O_2_-) treated ARPE-19 cells. 

## 2. Materials and Methods

### 2.1. Regents and Antibodies

Hydroquinone was obtained from Alfa Aesar A Jonson Matthey Company (USA). Analytical grade 30% H_2_O_2_ was obtained from Sinopharm Chemical Reagent Co., Ltd. (Shanghai, China) and was diluted with deionized water to the indicated concentrations for experiments. Lutein and zeaxanthin were from Shanghai Gongshuo Biotechnology Co., Ltd. (Shanghai, China). These two agents were dissolved in dimethyl sulfoxide (DMSO) in this study. The primers used in real-time PCR were from GenScript Co., Ltd. (Nanjing, China). The primary antibodies used in western blot analyses against cathepsin B (sc-6493), cystatin C (sc-73879), MMP-2 (sc-8835), and TIMP-2 (sc-21735) were from Santa Cruz Biotechnology (Santa Cruz, CA, USA). The primary antibody against *β*-actin (No. A2228) was from Sigma (St. Louis, MO, USA), and the horseradish peroxidase-conjugated secondary antibody (No. 3678) was from Cell Signaling Technology (Danvers, MA, USA).

### 2.2. Preparation of *Fructus lycii* Ethanol Extract

The herb *Fructus lycii *was collected from Ningxia Province (China) by the Pharmaceutical Preparation Section of Jiangsu Province Hospital of Traditional Chinese Medicine (Nanjing, China). The herb was soaked with 5 volumes of 60% ethanol overnight, then refluxed for 1 h and filtrated. The filter residues were extracted with 3 volumes of 60% ethanol under reflux for 1 h and filtrated. Two batches of filtrate were combined and concentrated and yielded viscous ethanol extract. Each gram of the ethanol extract contained 1.563 g crude herb.

### 2.3. Animals and Treatments

C57BL/6 mice (8-month-old female) were purchased from Beijing Vitalriver Experimental Animal Co., Ltd. (Beijing, China). All experimental procedures were approved by the institutional and local committee on the care and use of animals of Nanjing University of Chinese Medicine (Nanjing, China), and all animals received humane care according to the National Institutes of Health (USA) guidelines. All mice were maintained under a 12 h light/dark cycle at a controlled temperature (25°C) with free access to food and tap water. Forty mice were fed with high-fat diet for 6 months and subsequently received the intake of hydroquinone, which was dissolved in the drinking water, for additional 3 months. Afterwards, the 40 mice were divided into 4 groups (10 mice/each group), namely, AMD model group (high-fat diet plus hydroquinone intake), low dose group (1.25 g/kg treatment), moderate dose group (2.50 g/kg treatment), and high dose group (3.75 g/kg treatment). These doses corresponded to the crude drug doses used clinically, which have been sufficiently demonstrated to be safe in humans. The *Fructus lycii* ethanol extract was administrated to mice orally once a day for 3 months. During the course of experiments, ten mice were normally fed without being exposed to high-fat diet and hydroquinone, and they were termed “aging mice.” In addition, ten 3-month-old mice were used as negative control for histopathological examination, and they were termed “young mice” in this study.

### 2.4. Electron Microscopy Examination

After treatments, all mice were sacrificed, and eyeballs were isolated and fixed immediately in 2.5% glutaral plus 2.5% paraform for 24 h. Cornea, crystalline lens, and vitreous body were removed from the eye tissues. Wall tissue (2 × 4 mm) was excised from the bilateral area of optic disc and fixed with glutaral/osmic acid, coated with epoxy resins, and sectioned. Generally, the tissues were extracted from the back of the equator of eyeball. After double staining with uranyl acetate and lead citrate, the sections were examined with transmission electron microscope (JEM1010EX), and images were taken. The width of sediment beneath RPE was scored according to the following guidelines: 0 level, no sediment; 1 level, local spotted sediment; 2 level, the area of sediment <2 RPE cells; and 3 level, the area of sediment ≥2 RPE cells. The thickness of sediment beneath RPE was scored according to the following guidelines: 0 level, no sediment; 1 level, flat sediment; 2 level, the thickness <1/4 height of one RPE cell; and 3 level, the thickness ≥1/4 height of one RPE cell. The total score = width score + 1/2 thickness core [16]. The thickness of Bruch membrane was determined under electron microscopy directly (magnification ×25000). For all these examinations, every 5 sections from 1 eyeball were observed, and 3 random fields were visualized for scoring.

### 2.5. Immunohistochemical Evaluation

Cornea, lens, and vitrectomy were removed from the eye tissues. The wall of eyeball was divided into four pieces along with discus opticus. So the examined tissues could contain the superior, inferior, temporal, or nasal part. Tissue sections of 4 *μ*M were fixed with cold acetone for 10 min and washed with phosphate buffered saline (PBS) for 3 times. Then, slides were blocked with 10% goat serum for 10 min at room temperature followed by incubation with antibodies against cathepsin B (dilution 1 : 50) or cystatin C (dilution 1 : 50) overnight at 4°C. PBS was used as negative control. Slides were washed with PBS and then incubated with anti-rat IgG-HRP antibody for 30 min at 37°C followed by addition of chromogenic agent AEC. Finally, the slides were counterstained in hematoxylin and observed under an inverted microscope with a Leica Qwin System (Leica, Germany).

### 2.6. Cell Culture

Human RPE cell line ARPE-19 was purchased from the American Type Culture Collection (USA). ARPE-19 cells were cultured in Dulbecco's modified Eagle medium (DMEM; Invitrogen, Grand Island, NY, USA) supplemented with 10% fetal bovine serum (FBS; Sijiqing Biological Engineering Materials Co., Ltd., HangZhou, China), 100 U/mL penicillin, and 100 mg/mL streptomycin and grown in a 95% air and 5% CO_2_ humidified atmosphere at 37°C. 

### 2.7. MTS Assay

ARPE-19 cells in logarithmic growth were seeded in 96-well plates and cultured in DMEM supplemented with 10% FBS for 24 h and then treated with H_2_O_2_, with or without lutein/zeaxanthin at the indicated concentrations for 24 h. Treatment with DMSO (0.02%, w/v) was set up as the negative control, and this was performed throughout the *in vitro* study. After treatment, 3-(4,5-dimethylthiazol-2-yl)-5-(3-carboxymethoxyphenyl)-2-(4-sulfo-phenyl)-2H-tetrazolium (MTS; Sigma, St Louis, MO, USA) and phenazine methosulfate (Promega Corporation, Madison, WI, USA) were added, and the cells were further incubated for 3 h at 37°C. The spectrophotometric absorbance at 490 nm was measured by a SPECTRAmax microplate spectrophotometer (Molecular Devices, Sunnyvale, CA, USA). Six duplicate wells were set up for each group.

### 2.8. Real-Time PCR

Total RNA was isolated from retina and choroid using Trizol reagent (Sigma, St. Louis, MO, USA) following the protocol provided by the manufacturer. Real-time PCR was performed as described previously [17]. Glyceraldehyde phosphate dehydrogenase (GAPDH) was used as the invariant control. Fold changes in the mRNA levels of target genes related to the invariant control GAPDH were calculated as suggested by Schmittgen et al. [18]. The following primers were used in real-time PCR analyses: cathepsin B: (forward) 5′-GGAGATACTCCCAGGTGCAA-3′, (reverse) 5′-CTGCCATGATCTCCTTCACA-3′; cystatin C (forward) 5′-CAACAAGGGCAGCAACGATG-3′, (reverse) 5′-GGGAAGGAGCACAAGTAAGGAAC-3′; GAPDH: (forward) 5′-GGCCCCTCTGGAAAGCTGTG-3′, (reverse) 5′-CCGCCTGCTTCACCACCTTCT-3′.

### 2.9. Western Blot Analyses

The eye tissues or treated ARPE-19 cells were homogenised in RIPA lysis buffer (0.1% SDS, 0.5% deoxycholate, 1% Nonidet, 150 mM NaCl, and 50 mM Tris-HCl) containing protease inhibitors on ice. The protein levels were determined using a BCA assay kit (Pierce, USA). Proteins (50 *μ*g/well) were separated by SDS-polyacrylamide gel, transferred to a PVDF membrane (Millipore, Burlington, MA, USA), and blocked with 5% skim milk in Tris-buffered saline containing 0.1% Tween 20. Target proteins were detected by corresponding primary antibodies and subsequently by horseradish peroxidase-conjugated secondary antibodies. Protein bands were visualized using chemiluminescence reagent (Millipore, Burlington, MA, USA). Equivalent loading was confirmed using an antibody against *β*-actin. The levels of target protein bands were densitometrically determined using Quantity Ones 4.4.1 (Bio-Rad Laboratories, Berkeley, CA, USA). The variation in the density of bands was expressed as fold changes compared to the control in the blot after normalization to *β*-actin.

### 2.10. Statistical Analysis

Data were presented as mean ± SD, and results were analyzed using SPSS16.0 software. The significance of difference was determined by one-way ANOVA with the post hoc Dunnett's test. A value of *P* < 0.05 was considered to be statistically significant.

## 3. Results

### 3.1. Ethanol Extract of *Fructus lycii* Improves Histopathologic Changes in Mice with an Experimental Model of AMD

**Figure 1:**
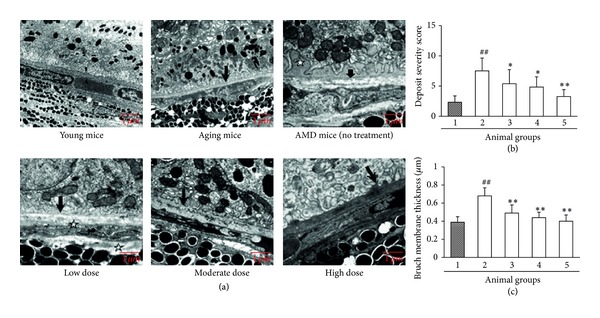
Effects of *Fructus lycii* ethanol extract on the histopathology in mice with an experimental model of AMD. Mice were grouped: group 1, aging control group (no high-fat diet plus hydroquinone, no treatment); group 2, AMD model group (with high-fat diet plus hydroquinone, no treatment); group 3, low dose group (with high-fat diet plus hydroquinone, 1.25 g/kg treatment); group 4, moderate dose group (with high-fat diet plus hydroquinone, 2.50 g/kg treatment); and group 5, high dose group (with high-fat diet plus hydroquinone, 3.75 g/kg treatment). (a) Electron microscopy examination of the eye tissues (magnification ×25000). Normal young mice (3 months old) were used as negative control. The black arrows indicate the Bruch membrane. (b) Comprehensive scoring on the severity of RPE sediment. The width and thickness of sediment were scored according to the guidelines described in Section 2. The total score = width score + 1/2 thickness score. Data are expressed as mean ± SD, ^##^ *P* < 0.01 versus group 1, *P < 0.05 versus group 2, and **P < 0.01 versus group 2. (c) Determination of Bruch membrane thickness. Data are expressed as mean ± SD, ^##^ *P* < 0.01 versus group 1, and **P < 0.01 versus group 2.

We used high-fat diet combined with hydroquinone intake to establish the AMD model in mice. Histopathological examination demonstrated that there was no RPE sediment in the young mice and less spotted sediment in the aging control mice, but massive successive flat sediment was observed in the AMD mice without treatment, suggesting that the AMD model was established successfully. However, treatment with *Fructus lycii* ethanol extract at various doses resulted in a reduction in RPE sediment (Figure 1(a)). These results were confirmed by the scoring for sediment severity. The score for the AMD mice without treatment was significantly higher than that for the aging control mice (*P* < 0.01), but *Fructus lycii* ethanol extract decreased the score in a dose-dependent fashion and at low dose led to a significant effect compared to the AMD mice without treatment (*P* < 0.05) (Figure 1(b)). Electron microscopy examination exhibited that the Bruch membrane of the aging control mice was slightly thicker than that of the young mice, while the Bruch membrane of the AMD mice without treatment was considerably thickened concomitant with loss of homogeneity. Similar results were also observed around the choroid capillary endothelial cells. However, *Fructus lycii* ethanol extract led to histological improvement in Bruch membrane to different degrees (Figure 1(a)) and significantly reduced the enhanced thickness of Bruch membrane in a dose-dependent manner compared to the AMD mice without treatment (all *P* < 0.01) (Figure 1(c)). Taken together, these data indicated that *Fructus lycii* ethanol extract could reduce RPE sediment and restore Bruch membrane* in vivo*.

### 3.2. Ethanol Extract of *Fructus lycii* Reduces Cathepsin B Expression in Mice with an Experimental Model of AMD

**Figure 2:**
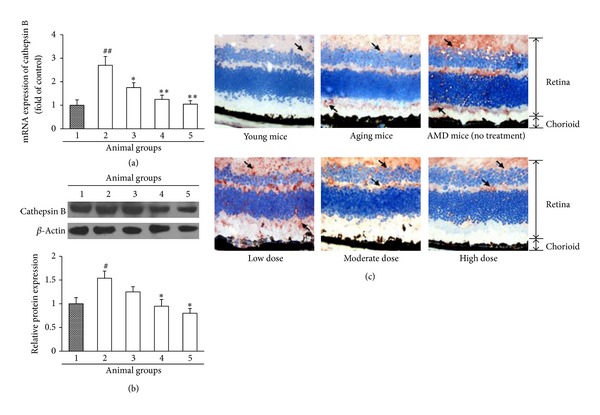
Effects of *Fructus lycii* ethanol extract on cathepsin B expression in mice with an experimental model of AMD. Mice were grouped: group 1, aging control group (no high-fat diet plus hydroquinone, no treatment); group 2, AMD model group (with high-fat diet plus hydroquinone, no treatment); group 3, low dose group (with high-fat diet plus hydroquinone, 1.25 g/kg treatment); group 4, moderate dose group (with high-fat diet plus hydroquinone, 2.50 g/kg treatment); and group 5, high dose group (with high-fat diet plus hydroquinone, 3.75 g/kg treatment). (a) Real-time PCR analysis for transcript level of cathepsin B. GAPDH was used as the invariant control for calculating fold changes in mRNA levels (*n* = 10). Data are expressed as mean ± SD, ^##^ *P* < 0.01 versus group 1, *P < 0.05 versus group 2, and **P < 0.01 versus group 2. (b) Western blot analysis for protein level of cathepsin B. *β*-Actin was used as an invariant control for equal loading. Representative blots were from 10 independent experiments with quantification. Data are expressed as mean ± SD, ^#^ *P* < 0.05 versus group 1, and *P < 0.05 versus group 2. (c) Immunohistochemical staining of the eye tissues for cathepsin B was detected in different groups (magnification ×25000). Normal young mice (3 months old) were used as negative control. The retina and chorioid are indicated.

We next examined whether the gene and protein expression of cathepsin B in the eye tissues was modulated by treatment with *Fructus lycii* ethanol extract. Real-time PCR showed that high-fat diet combined with hydroquinone intake led to a significant increase in the mRNA expression of cathepsin B compared to the young mice (*P* < 0.01), but this increase was dose-dependently abrogated by *Fructus lycii* ethanol extract. *Fructus lycii* ethanol extract at low dose produced a significant reducing effect compared to the AMD mice without treatment (*P* < 0.05) (Figure 2(a)). In addition, western blot assay and immunohistochemistry were used to determine the protein expression of cathepsin B. The data showed that the protein abundance of this enzyme was significantly elevated in the AMD mice without treatment, but treatment with *Fructus lycii* ethanol extract dose-dependently downregulated its expression in the eye tissues (Figures 2(b) and 2(c)). Collectively, these data indicated that the enhanced production of cathepsin B in the pathogenesis of AMD could be decreased by *Fructus lycii* ethanol extract at both gene and protein levels *in vivo*.

### 3.3. Ethanol Extract of *Fructus lycii* Reduces Cystatin C Expression in Mice with an Experimental Model of AMD

**Figure 3:**
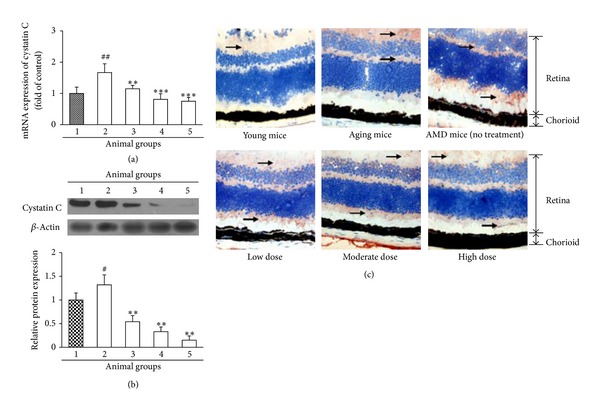
Effects of *Fructus lycii* ethanol extract on cystatin C expression in mice with an experimental model of AMD. Mice were grouped: group 1, aging control group (no high-fat diet plus hydroquinone, no treatment); group 2, AMD model group (with high-fat diet plus hydroquinone, no treatment); group 3, low dose group (with high-fat diet plus hydroquinone, 1.25 g/kg treatment); group 4, moderate dose group (with high-fat diet plus hydroquinone, 2.50 g/kg treatment); and group 5, high dose group (with high-fat diet plus hydroquinone, 3.75 g/kg treatment). (a) Real-time PCR analysis for transcript level of cystatin C. GAPDH was used as the invariant control for calculating fold changes in mRNA levels (*n* = 10). Data are expressed as mean ± SD, ^#^ *P* < 0.01 versus group 1, **P < 0.01 versus group 2, and ***P < 0.001 versus group 2. (b) Western blot analysis for protein level of cystatin C. *β*-Actin was used as an invariant control for equal loading. Representative blots were from 10 independent experiments with quantification. Data are expressed as mean ± SD, **P < 0.01 versus group 2. (c) Immunohistochemical staining of the eye tissues for cystatin C was detected in different groups (magnification ×25000). Normal young mice (3 months old) were used as negative control. The retina and chorioid are indicated.

Cystatin C as another important regulatory enzyme in AMD pathology was also determined in our present study. Similar to cathepsin B, the gene expression of cystatin C was also upregulated significantly in the AMD mice without treatment compared to the young counterparts (*P* < 0.01), but *Fructus lycii* ethanol extract dose-dependently eliminated the increase in cystatin C gene expression, and a significant effect was observed even if at a low dose compared to the AMD mice without treatment (*P* < 0.01) (Figure 3(a)). Furthermore, western blot assay and immunohistochemical analysis revealed that the protein expression pattern of cystatin C in the absence or presence of *Fructus lycii* ethanol extract was in accordance with the real-time PCR data (Figures 3(b) and 3(c)). Altogether, these findings suggested that *Fructus lycii* ethanol extract could inhibit the *de novo* production of cystatin C in mice, whose upregulation was related to the pathogenesis of AMD.

### 3.4. Lutein Combined with Zeaxanthin Stimulates Proliferation and Regulates Matrix Homeostasis in H_2_O_2_-Treated ARPE-19 Cells

**Figure 4:**
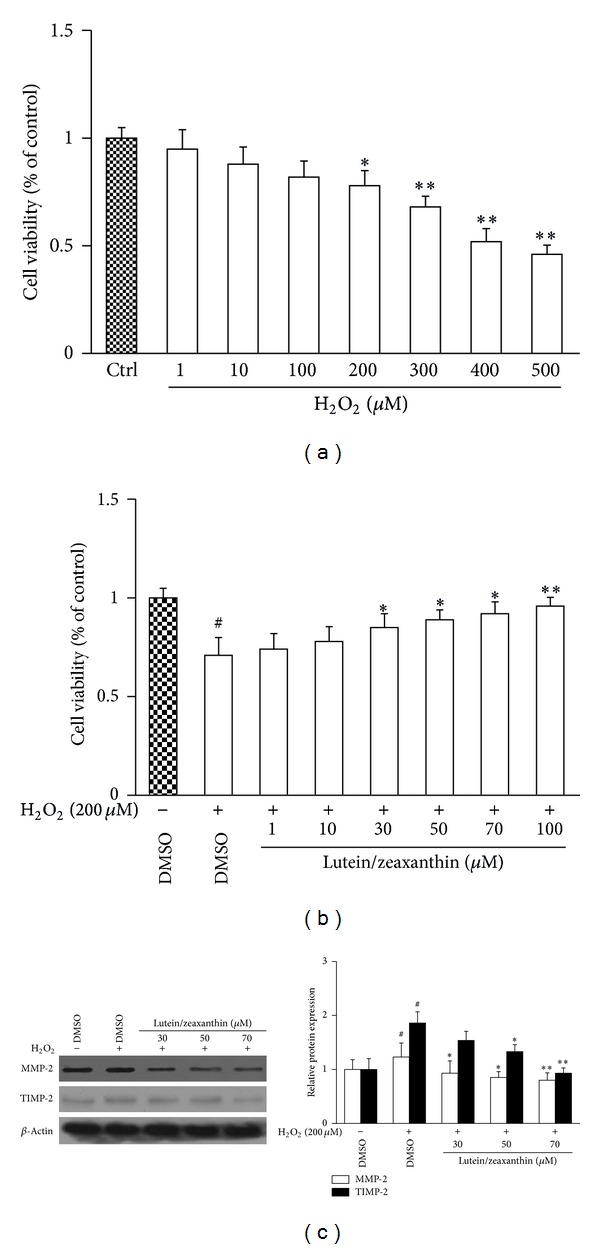
Effects of lutein/zeaxanthin on proliferation and MMP/TIMP system in ARPE-19 cells treated with H_2_O_2_. ARPE-19 cells were treated with H_2_O_2_, with or without lutein/zeaxanthin at the indicated concentrations for 24 h. Treatment with DMSO (0.02%, w/v) was set up as the negative control. (a) MTS assay for evaluation of cell viability. Data are expressed as mean ± SD, *P < 0.05 versus control, and **P < 0.01 versus control. (b) MTS assay for evaluation of cell viability. Data are expressed as mean ± SD, ^#^ *P* < 0.05 versus DMSO without H_2_O_2_, *P < 0.05 versus DMSO with H_2_O_2_, and **P < 0.01 versus DMSO with H_2_O_2_. (c) Western blot analysis for protein levels of MMP-2 and TIMP-2. *β*-Actin was used as an invariant control for equal loading. Representative blots were from three independent experiments with quantification. Data are expressed as mean ± SD, ^#^ *P* < 0.05 versus DMSO without H_2_O_2_, *P < 0.05 versus DMSO with H_2_O_2_, and **P < 0.01 versus DMSO with H_2_O_2_.

Lutein and zeaxanthin are two well-known carotenoid pigments found in *Fructus lycii*. They are isomers sharing the same molecular weight. We hypothesized that the two compounds could protect RPE cells due to their potent antioxidative properties and thereby had the potential to treat AMD. To test this hypothesis, we established an *in vitro* model of H_2_O_2_-treated ARPE-19 cells, which could mimic the oxidative stress-induced injury of RPE cells in the pathogenesis of AMD, and evaluated the protective effects of lutein combined with zeaxanthin (1 : 1) *in vitro*. Cell viability assay showed that H_2_O_2_ reduced ARPE-19 cell proliferation dose-dependently and produced a significant effect at 200 *μ*M compared to the control cells (*P* < 0.05) (Figure 4(a)). Therefore, we chose H_2_O_2_ at 200 *μ*M to establish the oxidative stress-induced injury of ARPE-19 cells. The subsequent experiment demonstrated that lutein/zeaxanthin reversed the H_2_O_2_-caused inhibition of cell viability in a dose-dependent manner. Lutein/zeaxanthin at 30 *μ*M produced a significant effect compared to the cells treated with H_2_O_2_ alone (Figure 4(b)). Further, we determined the protein expression of MMP-2 and TIMP-2 in ARPE-19 cells treated with H_2_O_2_ and/or lutein/zeaxanthin. The results revealed that H_2_O_2_ significantly upregulated the protein abundance of the two enzymes compared to the blank control cells (both *P* < 0.05), but lutein/zeaxanthin dose-dependently abolished the H_2_O_2_-induced increase in their expression (Figure 4(c)). These findings collectively demonstrated that lutein/zeaxanthin could restore the inhibited cell viability and modulate the MMP/TIMP system in ARPE-19 cells under H_2_O_2_-caused oxidative stress, suggesting a beneficial role for lutein/zeaxanthin in the antioxidative therapy for AMD.

## 4. Discussion

AMD is a complex, multifactorial disease of aging for which several theories of pathogenesis have been proposed, including oxidative damage and ocular perfusion abnormalities. Early AMD is clinically characterized by the formation of drusens and enhanced thickness of Bruch membrane [19]. Currently two hypotheses have been proposed to explain the mechanisms underlying the formation of sediment beneath RPE in AMD (1) Wound healing mechanism: phagocytosis of photoreceptor outer segment by RPE cells results in the formation of lipofuscin, whose oxidation produces a large amount of reactive oxygen species leading to damaged integrity of lysosomes, lipid peroxidation, and death of RPE cells. The undigested disk membrane and phagosomes are deposited between the RPE basilar membrane and Bruch membrane [20]. (2) Barrier mechanism: Bruch membrane with good permeability supports RPE cells and provides nutrition to the highly metabolic photoreceptor. Any changes in Bruch membrane structure or constituent may impair the diffusion properties leading to lipid deposition [21]. Epidemiological evidence has shown that high-fat diet, especially consumption of saturated fats, is associated with the incidence of AMD [22]. Recent animal studies demonstrated that genetic C57BL/6 mice with hyperlipemia did not show significant RPE sediment, but the sediment was considerably increased when combined with blue light exposure, suggesting that oxidative injury caused by light exposure was necessarily required for massive formation of RPE sediment [23]. Therefore, in our present study, we used high-fat diet plus hydroquinone intake to induce AMD in mice. We successfully established this model evidenced by the severe RPE sediment and enhanced Bruch membrane thickness in the AMD mice without treatment. Our results confirmed the notion that the retina is particularly susceptible to oxidative stress due to its high concentration of oxygen, its high proportion of polyunsaturated fatty acids, and its exposure to visible light and that this susceptibility also increases with aging in the macular region.

At present, there is no established way of preventing dry AMD. Using the established model, we investigated the effects of ethanol extract of *Fructus lycii*, a well-known nutritional herbal medicine, on AMD in mice and explored the preliminary mechanisms. Our results showed that the deposit severity and Bruch membrane thickness were all decreased significantly evidenced by histopathological examination, suggesting that *Fructus lycii* ethanol extract could protect mice from AMD induced by high-fat diet plus oxidative stress. Our findings also indicated that *Fructus lycii *has the potential to reduce the risk of progression of AMD. Increasing evidence reveals that cathepsin B and cystatin C are critically involved in the pathogenesis of AMD [24]. Cathepsin B is a broad spectrum lysosomal cysteine protease and plays an essential role in protein degradation. Wound healing process or inflammatory reaction may influence cathepsin B expression. Cystatin C is an inhibitor of cathepsin B and precisely in coordination with cathepsin B regulates protein synthesis and degradation [25]. Cathepsin B and cystatin C are primarily expressed in RPE cells. Studies have shown that cathepsin B in RPE cells is a major performer in degradation of membranous disc ablated from the top of outer segment of visual cells. Moreover, under oxidative stress both cathepsin B and cystatin C in RPE cells and choroid are upregulated, suggesting a tight correlation to the occurrence of AMD [26]. We therefore postulated that these two enzymes might be involved in the AMD model in mice and associated with the beneficial effects of *Fructus lycii *ethanol extract. Our data could support this postulation. The expression of cathepsin B and cystatin C was significantly increased at both gene and protein levels in mice with an experimental model of AMD, which further strengthened the association of these two enzymes with the development of AMD. *Fructus lycii* ethanol extract could suppress the gene and protein expression of cathepsin B and cystatin C. However, *Fructus lycii* ethanol extract showed a more significant inhibitory effect on cystatin C expression compared to cathepsin B, which could increase the ratio of cathepsin B/cystatin C, favoring cathepsin B to remove the RPE sediment and promoting recovery from AMD. These data were also consistent with the histopathologic observations by electron microscope. 

To further confirm the postulation that the beneficial effects of *Fructus lycii* ethanol extract on AMD were at least partially attributed to the antioxidant properties, we examined the *in vitro* effects of two carotenoid pigments lutein and zeaxanthin on ARPE-19 cells treated with H_2_O_2_, which could mimic the injury caused by oxidative stress in the pathogenesis of AMD. The carotenoids in the macular region can weaken the intensity of blue light and effectively attenuate the retinal oxidative stress. Previous studies demonstrated that lutein and zeaxanthin could reduce unsaturated fatty acid oxidation and inhibit apoptosis of photoreceptors via stabilizing the outer membrane [27]. A health and nutrition examination survey in the USA revealed that the incidence of early AMD in patients aged 40–59 years and advanced AMD in patients aged 60–79 years correlates tightly with the dietary consumption of lutein and zeaxanthin and suggested that dietary consumption of amounts of the two pigments could prevent the occurrence of early or advanced AMD [28]. Nevertheless, the molecular mechanisms underlying the anti-AMD properties of lutein and zeaxanthin remain to be elucidated. Our present studies preliminarily addressed this issue. We found that lutein/zeaxanthin rescued the inhibition of proliferation in H_2_O_2_-treated ARPE-19 cells, indicating that the two compounds could protect RPE cells against oxidative stress in the pathogenesis of AMD. Furthermore, studies have documented the association of MMP/TIMP system with AMD, indicating that the expression pattern of MMPs and/or TIMPs in RPE cells is altered shifting the balance of matrix components towards deposition [29]. MMP-2 is a gelatinase with proteolytic activity responsible for degradation of matrix and basement membrane leading to destruction of blood-retinal barrier. TIMP-2 can endogenously inhibit MMP activity at posttranscriptional level via forming MMP-TIMP complexes that block the binding of MMPs to substrates. Under normal conditions, MMP-2 and TIMP-2 are expressed coordinately and maintain the homeostasis of matrix components in eye tissues. In the pathogenesis of AMD, the equilibrium between MMP-2 and TIMP-2 is disrupted, promoting the progression of the disease [30, 31]. Our present data showed that these two molecules were all upregulated in ARPE-19 cells under oxidative stress. However, lutein/zeaxanthin abolished the elevated expression of MMP-2 and TIMP-2 in H_2_O_2_-treated ARPE-19 cells, suggesting that lutein/zeaxanthin could be beneficial for oxidative stress-involved AMD by regulating matrix homeostasis. Our *in vitro* findings were essentially complimentary to the *in vivo *data on the anti-AMD properties of *Fructus lycii* ethanol extract. Composition analysis has shown that carotenoids, betaine, and polysaccharide are the major bioactive substances in *Fructus lycii*. Therefore, it would be interesting to investigate whether other components apart from carotenoids could be beneficial for AMD.

In summary, our results demonstrated that *Fructus lycii* ethanol extract effectively reduced the RPE sediment and Bruch membrane thickness in mice with an experimental model of AMD induced by high-fat diet combined with hydroquinone intake. These beneficial effects might be associated with the decreased expression of cathepsin B and cystatin C at both gene and protein levels. Investigations* in vitro* showed that lutein/zeaxanthin stimulated proliferation and downregulated the expression of MMP-2 and TIMP-2 in H_2_O_2_-treated ARPE-19 cells. Our results strongly suggested that *Fructus lycii *as a beneficial dietary herb might reduce the risk of progression of AMD.

## Abbreviations

AMD: Age-related macular degeneration
DMEM: Dulbecco's modified eagle medium
FBS: Fetal bovine serum
GAPDH: Glyceraldehyde phosphate dehydrogenase
H_2_O_2_: Hydrogen peroxide
MMPs: Matrix metalloproteinases
MTS: 3-(4,5-Dimethylthiazol-2-yl)-5-(3-carboxymethoxyphenyl)-2-(4-sulfo-phenyl)-2H-tetrazolium
PBS: Phosphate buffered saline
RPE: Retinal pigment epithelium
TIMPs: Tissue inhibitor of metalloproteinases.
